# Distribution pattern of left-ventricular myocardial strain analyzed by a cine MRI based deformation registration algorithm in healthy Chinese volunteers

**DOI:** 10.1038/srep45314

**Published:** 2017-03-28

**Authors:** Hong Liu, Dan Yang, Ke Wan, Yong Luo, Jia-Yu Sun, Tian-Jing Zhang, Wei-Hao Li, Andreas Greiser, Marie-Pierre Jolly, Qing Zhang, Yu-Cheng Chen

**Affiliations:** 1 Cardiology Division, West China Hospital, Sichuan University, Chengdu, Sichuan Province, China; 2 Radiology Department, West China Hospital, Sichuan University, Chengdu, Sichuan Province, China; 3 Siemens Healthineers Northeast Asia Collaboration, Beijing, China; 4 Siemens Healthineers, Erlangen, Germany; 5 Siemens Healthineers, Medical Imaging Technologies, Princeton, NJ, USA

## Abstract

The cine magnetic resonance imaging based technique feature tracking-cardiac magnetic resonance (FT-CMR) is emerging as a novel, simple and robust method to evaluate myocardial strain. We investigated the distribution characteristics of left-ventricular myocardial strain using a novel cine MRI based deformation registration algorithm (DRA) in a cohort of healthy Chinese subjects. A total of 130 healthy Chinese subjects were enrolled. Three components of orthogonal strain (radial, circumferential, longitudinal) of the left ventricle were analyzed using DRA on steady-state free precession cine sequence images. A distinct transmural circumferential strain gradient was observed in the left ventricle that showed universal increment from the epicardial to endocardial myocardial wall (epiwall: −15.4 ± 1.9%; midwall: −18.8 ± 2.0%; endowall: −22.3 ± 2.3%, P < 0.001). Longitudinal strain showed a similar trend from epicardial to endocardial layers (epiwall: −16.0 ± 2.9%; midwall: −15.6 ± 2.7%; endowall: −14.8 ± 2.4%, P < 0.001), but radial strain had a very heterogeneous distribution and variation. In the longitudinal direction from the base to the apex of the left ventricle, there was a trend of decreasing peak systolic longitudinal strain (basal: −23.3 ± 4.6%; mid: −13.7 ± 7.3%; apical: −13.2 ± 5.5%; P < 0.001). In conclusion, there are distinct distribution patterns of circumferential and longitudinal strain within the left ventricle in healthy Chinese subjects. These distribution patterns of strain may provide unique profiles for further study in different types of myocardial disease.

Myocardial strain is an important parameter used to quantify myocardial deformation. Three principal components of strain (radial, circumferential and longitudinal) can be measured, and each component used to reflect different ways of impairment in cardiac function^1^,^2^. Echocardiography was the primary imaging modality to detect myocardial strain due to easy availability and convenience. However, the angle dependence of tissue Doppler ultrasound, acoustic-window limitation, and operator dependency limit application of echocardiographic imaging of myocardial strain in a clinical setting. Cardiovascular magnetic resonance (CMR) “tagging” has been considered to be the “gold standard” for analyses of myocardial strain^3^,^4^. Application of this method requires an additional sequence for data acquisition and post-processing analyses are time-consuming. Feature tracking-cardiovascular magnetic resonance (FT-CMR) is emerging as a promising post-processing method for analyses of myocardial strain^5^. FT-CMR has shown comparable results with CMR tagging^6^,^7^,^8^. More importantly, FT-CMR analyses are based on balanced standard steady-state free-precession (bSSFP) cine sequences instead of additionally acquired sequences. Feature tracking algorithm is based on the tracking of limited points on manually drawn cardiac endo- and epicardial contours. Cine MRI based deformation registration algorithm (DRA) (software name: TrufiStrain, Siemens Healthcare, Medical Imaging Technologies, Princeton, NJ, USA) is a novel technique for analysis of strain. DRA allows myocardial strain on a pixel basis, which was recently demonstrated to have superior reproducibility compared with FT-CMR, and to have better correlation with speckle-tracking echocardiography^9^.

Recently, normal reference values of FT-CMR for a European population were published but those of non-European descents^10^,^11^, such as for a Chinese population, have not. Due to the possible difference of cardiac size and function among different ethnicities, it is very important to obtain the reference value of cine bSSFP based myocardial strain in a healthy Chinese population, which will benefit future studies in the very large Chinese population worldwide. The left ventricular wall comprises endocardial, mid myocardial and epicardial layers. Myocardial fibers array in unique ways in different layers, which could impact the strain distribution in the heart. On the other hand, the orientation of myocardial fibers varies at different levels (base, mid ventricle and apex) along the long axis of the left ventricle (LV). The complicated organization of myocardial fibers constitutes very sophisticated left ventricular mechanics, strain, torsion and twisting. Other than the well-known importance of global strain, layer-specific strain and distribution patterns of regional strain have not been investigated. Analyses of multiple-layer strain by echocardiography have been developed, and a transmural strain gradient demonstrated^12^,^13^,^14^. However, layer-specific analyses of myocardial strain by FT-CMR have not been studied. In addition, whether a cine MRI based technique can demonstrate a pattern of strain distribution in the heart is not known. DRA (TrufiStrain) is pixel based analysis software^15^,^16^ that can provide layer-specific (endo/mid/epiwall) analyses of myocardial strain. In the present study, we explored the normal reference values of layer-specific strain and distribution characteristics in the LV using TrufiStrain in a large cohort of healthy Chinese volunteers. Furthermore, the effect of sex and age on distribution of strain was also investigated.

## Results

### Demographic data, volume and function of the LV

A total of 130 healthy volunteers aged 47 ± 17 (range, 18–83) years were enrolled (Table 1). Sixty participants (46.2%) were males. All of the images were tracked adequately.

### Mean global strain, mean layer-specific strain and transmural strain distribution of three orthogonal strains

Global mean systolic strain for radial strain (Err) was 38.8 ± 7.3%, for circumferential strain (Ecc) was −18.5 ± 2.0%, and for longitudinal strain (Ell) was −15.4 ± 2.4%. Table 2 shows the layer-specific strains. There was an obvious transmural gradient of Ecc within the LV myocardial wall (epiwall: −15.4 ± 1.9%; midwall: −18.8 ± 2.0%; endowall: −22.3 ± 2.3%; P < 0.001 between either two groups, respectively). Transmural distribution of Err was heterogeneous and showed wide variability. Mean endocardial peak systolic Err was significantly lower than that in the mid-myocardium or epicardium (P < 0.001), but no significant difference was present between the midcardial layer and epicardial layer (P = 0.15). Ell in the endocardial layer was slightly higher than that in the epicardial layer (endowall −16.0 ± 2.9%; midwall: −15.6 ± 2.7%; epiwall: −14.8 ± 2.4%; P < 0.001).

### Characteristics of strain distribution on the long axis from the LV base to the apex

Table 2 also shows the longitudinal distribution characteristics of Err, Ecc and Ell from the base to the apex of the LV. Err at the mid-LV level was the highest compared with at the base and apex (basal: 38.8 ± 8.5%; mid: 42.5 ± 8.6%; apical: 35.2 ± 10.6%; P < 0.001). Ecc was highest at the apical level compared with basal or mid-LV levels (basal: −17.6 ± 1.8%, mid: −16.8 ± 1.9%, apical: −21.0 ± 3.6%; P < 0.001). Peak systolic Ell showed a distinct decreasing pattern from base to apex (basal: −24.0 ± 4.0%; mid: −14.6 ± 5.4%; apical: −13.2 ± 3.5%; P < 0.001).

### Sex- and age-related strain distribution

There was some difference in Ecc and Ell values in different myocardial layers between females and males. In general, female volunteers had a greater value of layer-specific Ecc or Ell than males (Table 3). However, a transmural gradient of circumferential strain was present for both sexes. When evaluated by different levels from the base to the apex, Ecc and Ell were also higher in females than in males except at the apex (Table 4). Ell distribution in the longitudinal direction also decreased from the base to the apex for both sexes.

Age had only a mild-to-moderate correlation with some strain parameters. Peak systolic Err in different layers showed modest correlation with age (endowall: r = 0.41, P < 0.001; midwall: r = 0.42, P < 0.001; epiwall: r = 0.39, P < 0.001). Peak systolic Ecc was also correlated slightly with age in the endowall (r = 0.32, P < 0.001) and midwall (r = 0.24, P = 0.01). Also, peak systolic Ell had a mild correlation between age and epicardial strain values (r = 0.17, P = 0.048). There was no significant correlation between other layer-specific strain values and age (Ecc: epiwall r = 0.07, P = 0.41; Ell: endowall r = 0.16, P = 0.07; midwall r = 0.17, P = 0.05).

### Transmural gradient of circumferential strain (G_endo-epi_) and longitudinal gradient of longitudinal strain (G_base-apex_)

**Figure 1:**
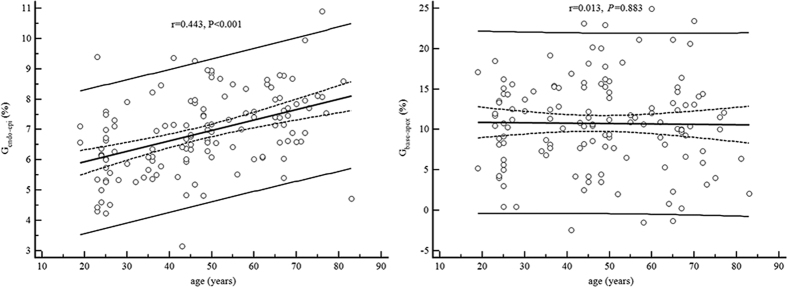
Age-related systolic strain values of G_base-apex_ and G_endo-epi_. Scatter diagrams with regression lines include the regression line with the respective 95% confidence curves as well as the 95% prediction curves. G_base-apex_: longitudinal gradient from the base to the apex of longitudinal strain. G_endo-epi_: transmural gradient from the endowall to the epiwall of circumferential strain.

Mean G_endo-epi_ in all subjects was 6.9 ± 1.3% (Table 5), and no significant difference was seen between sexes (P > 0.05). Linear regression analyses revealed age (r = 0.44, P < 0.001), weight (r = 0.18, P = 0.04), body mass index (r = 0.36, P < 0.001), systolic blood pressure (r = 0.27, P = 0.003) and heart rate (r = 0.21, P = 0.02) to be associated significantly with G_endo-epi_. However, multivariate linear regression analyses showed only the body mass index (P < 0.001) and age (P < 0.001) to be correlated independently with G_endo-epi_ (Fig. 1). Sex, age or other demographic data had no effect on the Ell longitudinal gradient.

### Inter-observer and intra-observer variability for strain analyses

The intra-observer agreement of the three principal strain measurements was excellent (Table 6), with the CVs ranging from 1% to 4% and ICCs > 0.85 at three layers. For inter-observer agreement, the variability was small, with CVs ranging from 3% to 6% and ICCs > 0.83 at three layers.

## Discussion

This is the first study to demonstrate the distribution pattern of strain of the left ventricle in a large cohort of healthy subjects using a novel cine MRI based DRA technique (TrufiStrain). Our study elicited three main findings. First, there was a distinct transmural gradient of Ecc in the LV myocardium in which endocardial Ecc was the highest and epicardial was the lowest. Second, Ell in the left ventricle decreased from the base to the apex. Finally, the distribution pattern was independent of sex and age, but the transmural gradient of Ecc was correlated significantly with age.

Compared with speckle-tracking echocardiography (STE), FT-CMR provides robust analyses for myocardial strain due to the high spatial resolution and good image quality^17^. Studies have shown that >95% of segments can be analysed accurately by CMR, whereas a significant amount of segments cannot be analyzed by STE due to impaired image quality^18^,^19^,^20^. Therefore, FT-CMR can be considered to be a robust method for strain analyses^6^,^21^. Recent studies have demonstrated FT-CMR to be independent of scanner vendors and field strength^16^,^17^,^18^,^22^. So far, almost all studies have been based on the widely used Tomtec software. DRA (Trufistrain) is novel software used to calculate myocardial strain based on cine SSFP images. DRA is a pixel-wise analysis of deformation, which is different to Tomtec software in which some points on endocardial or epicardial contours were tracked. Due to pixel-wise analysis of deformation, it is possible to divide all myocardial pixels into three myocardial layers, whereas classic feature tracking provides only endocardial or epicardial layers. Because of differences in the calculation algorithm, differences in strain value between software packages should be considered. One study compared the differences and variance between feature-tracking software (Tomtec imaging system, 2D CPA MR, Cardiac Performance Analysis, Version 1.1.2.36) and tissue-tracking software (Circle Cardiovascular Imaging, Tissue Tracking, cvi42)^23^, in which a significant difference in the strain value was found. Recently, a study compared TrufiStrain and Tomtec, and results showed TrufiStrain to have better correlation with strain derived from echocardiography. In addition, TrufiStrain had better reproducibility than FT-CMR^9^. Normal reference values in a European population were published recently, and global strains were also provided as a clinical reference^10^,^11^. The present study comprised 130 healthy Chinese participants and provided normal reference values for this novel strain analysis technique. The global peak systolic strain in three orthogonal directions in the left ventricle was provided, and some discrepancy was found compared with other publications, which could be explained by the use of different algorithms.

Global strain is usually employed for evaluating cardiac function and the risk of cardiovascular disease. Layer-specific strain has not been studied widely due to a limitation in methods. Clark and colleagues were the first to report the endocardial-to-epicardial Ecc gradient using tagging with 1.5-T MRI in 10 healthy hearts^24^. Similar findings were confirmed by STE, tissue Doppler imaging (TDI), phase-contrast magnetic resonance (PC-MR), and strain-encoded (SENC) CMR^12^,^13^,^25^. Most studies carried out transmural analyses by dividing the myocardial wall into two layers (endocardial and epicardial)^10^,^26^,^27^,^28^. Recently invented multiple-layer STE can be used to analyze strain in three myocardial layers, which could reflect layer-specific strains in accordance with anatomic myocardial fiber layers (endo/mid/epiwall)^12^. To date, layer-specific strain derived from cine-CMR has not been assessed. This knowledge gap could be explained by a lack of multiple-layer software or concern about accuracy and reproducibility. In our study, TrufiStrain software provided the chance to analyze multiple-layer strain. In our study, there was a very clear Ecc gradient within the LV myocardial wall, and this result was in accordance with previous results using other methods. Compared with a distinct transmural gradient in the LV myocardial wall, layer-specific Err by TrufiStrain did not show a consistent gradient in the present study. Strain studies using echocardiography have shown Err to increase from epicardial to endocardial layers^12^,^13^,^29^, whereas displacement encoding with stimulated echoes (DENSE) studies demonstrated controversial results^30^. Err measured by different methods manifested wide variability, and a similar variability was found in our study^10^,^23^. This variability could explain the inconsistency among the different methods used. Layer-specific Ell in our study showed an increasing trend from epicardial to endocardial walls, a result that is in accordance with other studies^11^,^14^,^21^,^26^. Only a few studies have observed strain-distribution patterns from the base to the apex of the LV. In the present study, the Ell distribution pattern decreased from the base to the apex of the LV. This characteristic had been shown by echocardiography-based studies^6^,^31^,^32^. Ecc from the base to the apex of the LV also had an increasing trend, and the apical level was the highest. This phenomenon has been observed previously^14^,^26^. However, Err at different levels of the left ventricle also did not show a homogenous result.

The transmural gradient of Ecc may contribute to better understanding of the physiologic and pathologic characteristics of the heart. Initial studies using FT-CMR showed a sex-based difference in strain parameters, and age was also associated with strain values^10^,^11^. In the present study, the distribution pattern (transmural or from the LV base to apex) was not dependent upon sex. Layer-specific strain analyses and the distribution pattern could also provide sensitive and early detection for some heart diseases. Bachner-Hinenzon *et al*. reported that endocardial peak systolic Ecc provides a better description than global strain in the myocardial wall at the acute stage of myocardial infraction in rats^33^. Okada and colleagues reported that, in patients with hypertrophic cardiomyopathy, a reduced Ecc in the mid and outer layers perhaps reflects impairment of a specific layer of the myocardium^2^. It is possible that the transmural gradient may be a unique parameter of myocardial diseases. In an echocardiography-based study, transmural strain gradient was established as a new parameter to evaluate wall motion, and was considered to be a more robust parameter of strain than global strain^34^. Therefore, in the present study, a parameter named G_epi-endo_ in Ecc was also calculated, which showed an interesting association with age. Whether this parameter could be used in evaluation of cardiac function in different types of cardiovascular diseases needs further research. The distribution pattern of Ell from the base to the apex of the LV would also have clinical significance. In an animal study of tachycardia-induced cardiomyopathy, abnormality in the strain distribution pattern on long-axis view was observed in a disease group compared with a normal control group^35^. In our study, the longitudinal gradient of Ell was independent of sex, age and other demographic parameters, which suggested that this gradient could be very stable in a healthy population. So far, very few studies have focused on the distribution pattern of strain in the longitudinal direction, and this phenomenon in clinical situations needs more validation and exploration.

Reproducibility was a major concern in previous FT-CMR studies. Most of the studies demonstrated that global strain had lower variability than segment-based analyses. In the present study, analyses of the distribution pattern were based on layer-specific strain, and not based on segments. This reduced variability based on each segment provided robust and stable analyses for strain distribution in the LV myocardial wall. In addition, the novel pixel-wise algorithm has been demonstrated to have better reproducibility than traditional FT-CMR. Our results also showed very good inter- and intra-observer variability in layer-specific analyses using this new FT-CMR software.

The main limitation of the present study was that TrufiStrain is new software based on cine-MRI images. As such, it has not been validated with the traditional tagging method, though it has been validated with Tomtec software and STE.

## Conclusions

DRA (TrufiStrain software) can be used to quantitatively analyze deformation of the LV myocardium, and provides the parameters of layer-specific strain. A distinct distribution pattern for transmural Ecc and Ell was observed. These unique patterns provide more information for evaluation of cardiac function, and could be new parameters used to identify the pathophysiologic basis of different types of heart disease.

## Methods

### Ethics statement

The study protocol was approved by the institutional review board of West China Hospital (Chengdu, China). All volunteers provided written informed consent to participate in this study, which was conducted in accordance with approved guidelines.

### Study population

Between June 2013 and December 2014 we enrolled 130 healthy adult volunteers without known cardiovascular diseases. All subjects received detailed history-taking, physical examination, 12-lead electrocardiography, blood pressure measurements, blood tests (including complete blood count, liver and renal function tests) and transthoracic echocardiography screening. Inclusion criteria were age >18 years without any known: cardiovascular disease; hypertension; cerebrovascular disease; nervous system disease; chronic lung disease; diabetes mellitus; cancer; autoimmune diseases; recent (within 1 month) systemic infection; recent (within 1 month) surgical procedure or severe trauma; taking of medications recently; history of implantation of a pacemaker or other metal object that could be a contraindication for MRI. Subjects with abnormal findings on the comprehensive examination, including a resting blood pressure ≥140/90 mmHg, abnormal complete blood count, liver/renal function tests, abnormal ECG, or abnormal findings by echocardiography screening, were excluded. Claustrophobia, or intolerance to breath-holding during CMR scanning, or poor image quality due to remarkable artifacts such as banding artifacts, were also excluded.

### CMR acquisition

Data were acquired using a 3.0-T MR scanner (Magnetom Trio; Siemens Healthineers, Erlangen, Germany) equipped with a four-element phased-array body coil. Images were acquired during breath-holds with electrocardiographic gating. bSSFP cine images were acquired in three long axes (two-, three-, and four-chamber) and consecutive short-axis views covering the LV from base to apex (repetition time (TR) 3.4 ms; echo time (TE) 1.3 ms; flip angle, 50°; field of view, 340 × 480 mm; matrix size, 256 × 144; slice thickness, 8 mm). Temporary resolution was 42 ms and spatial resolution was 1.3 × 1.5 mm, and 25 cardiac frames were reconstructed in each cardiac cycle.

### Image analyses

#### Volume and function of the left ventricle

CMR images were analyzed using dedicated cardiac post-procession software (Qmass 7.6; Medis Medical Imaging Systems, Leiden, the Netherlands). LV volume was measured by manually tracing the endocardial and epicardial borders at end-diastole and end-systole on successive short-axis cine images. Papillary muscle and trabeculations were included in the blood pool. Ventricular volume was calculated by volume summation in consecutive short-axis slices. Left-ventricular ejection fraction (LVEF) was calculated as: LVEF = (Volume end-diastole − volume end-systole)/(volume end diastole), All volume data were indexed as the ratio to body surface area.

#### FT-CMR strain analyses

**Figure 2:**
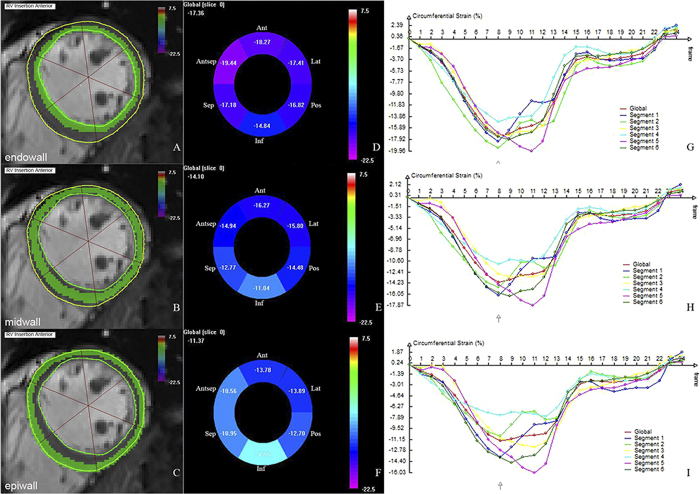
Deformable registration algorithm (DRA) based analysis (mid level) from the short-axis of the left ventricle. Ant = anterior wall; Antsep = anteroseptal wall; Sep = inferoseptal wall; Inf = inferior wall; Post = posterior wall; Lat = anterolateral wall. The endocardial and epicardial borders were traced manually at the end-diastole frame in the mid-slice cine image. The software tracked the contour throughout the cardiac cycle automatically and divided the myocardium into three layers (**A**, endowall; **B**, midwall; **C**, epiwall). Strain values of six segments are shown in the “Bull’s eye” (**D**,**E**,**F**). Strain curves of six segments and global myocardium are displayed on the right (**G**,**H**,**I**).

Strain analyses were done using DRA (TrufiStrain, Siemens Healthcare, Medical Imaging Technologies, Princeton, NJ, USA) (Fig. 2). Radial or circumferential strain was quantified on short-axis view at the basal, mid-ventricular and apical level of the LV. All slices were chosen at end-diastole. The basal level should be the first slice that excludes the outflow tract in end-systolic and diastolic phases. The mid slice of the LV was defined as the middle level. The apical level was the midpoint between the apex slice and mid slice. Longitudinal strain was quantified on a standard four-chamber long-axis view. LV endocardial and epicardial contours were drawn manually at end-diastole and then consecutive contours on the other phases through the cardiac cycle were tracked automatically by TrufiStrain. A segmentation algorithm was allowed to access the deformation from any frame to any other frame in the cardiac sequence^36^,^37^. The software divided the myocardium into three myocardial layers (endo/mid/epiwall). To define three layers, the software defined a line at 33.3% of the myocardium, and another line at 66.6%. The endowall was defined by all pixels falling between the endocardium and the 33.3% line. The mid wall layer was defined by all pixels located between the 33.3% line and 66.6% line, and the epiwall was defined between the epicardium and the 66.6% line. In the present study, peak systolic strain (Err/Ecc/Ell) of the entire myocardium and specific layers were included in analyses. Global mean myocardial and layer-specific strains (endo/mid/epiwall) were calculated by the mean value at base, mid and apical levels. The value of Gbase-apex was presented as the longitudinal gradient from the base to the apex of longitudinal strain, and Gendo-epi was for the transmural gradient from the endowall to the epiwall of circumferential strain.

### Reproducibility

Inter- and intra-observer variability for measurement of strains was assessed by repeated analyses of 26 (20% of all subjects) participants selected randomly. For assessment of the reproducibility of intra-observer agreement, tracing and analyses were done twice by an experienced operator (H.L., with 3 years’ experience), and the interval between the two analyses was ≥14 days. Inter-observer variability (D.Y., with 3 years’ experience), was obtained by two independent observers blinded to each other evaluating the same subjects.

### Statistical analyses

Continuous variables are expressed as the mean ± standard deviation (SD). The Shapiro–Wilk test was employed to check the normal distribution of continuous variables. Differences between genders were compared using the independent-sample *t*-test. Strain measurements from the three levels or layers were compared using one-way repeated analysis of variance (ANOVA) with a Tukey’s *post hoc* test for multiple comparisons (normally distributed variables) or Friedman test (non-parametric variables). Linear regression was used to analyze the relationship between myocardial strain and age. Reproducibility was assessed with Bland–Altman plots, interclass correlation coefficient (ICC) and coefficient of variation (CV). Statistical analyses were done using SPSS v19.0 (IBM, Armonk, NY, USA) and MedCalc v13.0 (MedCalc, Ostend, Belgium). P < 0.05 was considered significant.

## Additional Information

**How to cite this article**: Liu, H. *et al*. Distribution pattern of left-ventricular myocardial strain analyzed by a cine MRI based deformation registration algorithm in healthy Chinese volunteers. *Sci. Rep.* **7**, 45314; doi: 10.1038/srep45314 (2017).

**Publisher's note:** Springer Nature remains neutral with regard to jurisdictional claims in published maps and institutional affiliations.

## Figures and Tables

**Table 1:** Baseline characteristics and parameters of CMR function of normal subjects.

	Total	Female	Male
Number (%)	130 (100%)	70 (53.8%)	60 (46.2%)
Age (years)	47.3 ± 16.9	48.1 ± 16.7	46.5 ± 17.2
18–40	45 (34.6%)	22 (16.9%)	23 (17.7%)
41–65	58 (44.6%)	34 (26.2%)	24 (18.5%)
66–83	27 (20.8%)	14 (10.8%)	13 (10%)
Height (cm)	160.3 ± 8.7	154.5 ± 5.0*	167.1 ± 7.2
Weight (kg)	58.2 ± 8.9	52.9 ± 6.5*	64.4 ± 7.2
BMI (kg/m^2^)	22.6 ± 2.5	22.2 ± 2.6*	23.1 ± 2.4
BSA (m^2^)	1.6 ± 0.2	1.5 ± 0.1*	1.7 ± 0.1
SBP (mmHg)	118.5 ± 9.9	115.5 ± 9.2*	122.0 ± 9.6
DBP (mmHg)	77.8 ± 8.7	75.8 ± 8.7*	80.1 ± 8.5
HR (bpm)	73.4 ± 8.6	74.3 ± 8.4	72.3 ± 8.5
LVEDV (mL)	117.1 ± 25.2	104.2 ± 16.8*	132.2 ± 25.2
LVEDVi (ml/m^2^)	72.8 ± 11.5	69.0 ± 6.7*	76.5 ± 12.2
LVESV (mL)	40.8 ± 13.0	35.3 ± 8.6*	47.1 ± 14.4
LVESVi (mL/m^2^)	25.2 ± 6.6	23.6 ± 5.4*	27.2 ± 7.4
LVSV (mL)	76.4 ± 15.0	68.9 ± 11.2*	85.1 ± 14.2
LVMass (g)	79.7 ± 19.2	67.4 ± 9.9*	94.1 ± 17.5
LVMassi (g/m^2^)	49.3 ± 8.3	45.0 ± 5.3*	54.4 ± 8.3
LVEF (%)	65.9 ± 6.2	66.3 ± 5.1	65.4 ± 7.2

Values are the mean ± SD. *Male vs. female P < 0.05.

BMI = body mass index; BSA = body surface area; SBP = systolic blood pressure; DBP = diastolic blood pressure; HR = heart rate; LV = left ventricle; EDV = end-diastolic volume; EDVi = end-diastolic volume index; ESV = end-systolic volume; ESVi = end-systolic volume index; SV = stroke volume; Massi = mass index; EF = ejection fraction.

**Table 2:** Three-layer and three-level strain values in 130 healthy volunteers.

	Layer			Level		
	Endowall	Midwall	Epiwall	Basal	Mid	Apical
Err	34.2 ± 7.3^*,#^	41.7 ± 8.1	43.1 ± 7.9	38.8 ± 8.5^¥,^	42.4 ± 8.6^$^	35.2 ± 10.6
Ecc	−22.3 ± 2.3^*,#^	−18.8 ± 2.0^&^	−15.4 ± 1.9	−17.6 ± 1.8^¥,^	−16.8 ± 1.9^$^	−21.0 ± 3.6
Ell	−16.0 ± 2.9^#^	−15.6 ± 2.7^&^	−14.8 ± 2.4	−24.0 ± 4.0^¥,^	−14.6 ± 5.4^$^	−13.2 ± 3.5

Strain values are given in % and presented as the mean ± SD.

Err = radial strain; Ecc = circumferential strain; Ell = longitudinal strain.

^*^Significant difference between the endowall and midwall, p < 0.001.

^#^Significant difference between the endowall and epiwall, p < 0.001.

^&^Significant difference between the midwall and epiwall, p < 0.001.

^¥^Significant difference between the base and mid, p < 0.05.

Significant difference between the base and apex, p < 0.05.

^$^Significant difference between the mid and apex, p < 0.05.

**Table 3:** Sex-specific strain values of different myocardial layers in orthogonal directions.

	Endowall			Midwall			Epiwall		
	Male (n = 60)	Female (n = 70)	P (M vs. F)	Male (n = 60)	Female (n = 70)	P (M vs. F)	Male (n = 60)	Female (n = 70)	P (M vs. F)
Err	34.0 ± 7.7	39.5 ± 7.3	0.759	41.4 ± 8.2	42.0 ± 8.0	0.669	41.8 ± 7.6	44.2 ± 8.0	0.085
Ecc	−21.9 ± 2.2	−22.6 ± 2.3	0.074	−18.3 ± 1.9	−19.3 ± 2.1	0.010	−14.8 ± 1.7	−15.9 ± 1.9	<0.001
Ell	−14.8 ± 1.7	−17.2 ± 2.5	<0.001	−14.4 ± 1.8	−16.6 ± 2.5	<0.001	−13.7 ± 1.7	−15.7 ± 2.4	<0.001

Strain values are given in % and presented as the mean ± SD.

Abbreviations are as denoted in Table 2.

**Table 4:** Sex-specific strain values of different myocardial levels in orthogonal directions.

	Basal			Mid			Apical		
	Male (n = 60)	Female (n = 70)	P (M vs. F)	Male (n = 60)	Female (n = 70)	P (M vs. F)	Male (n = 60)	Female (n = 70)	P (M vs. F)
Err	37.5 ± 8.4	40.0 ± 8.5	0.092	41.9 ± 9.2	42.9 ± 8.1	0.490	34.8 ± 10.4	35.6 ± 10.9	0.667
Ecc	−17.2 ± 1.8	–17.9 ± 1.8	0.015	−16.2 ± 1.8	−17.4 ± 1.7	<0.001	−20.4 ± 3.5	−21.6 ± 3.6	0.058
Ell	−23.0 ± 3.5	−24.8 ± 4.3	0.012	−12.0 ± 4.6	−16.8 ± 5.1	<0.001	−12.8 ± 3.5	−13.6 ± 3.5	0.206

Strain values are given in % and presented as the mean ± SD.

Abbreviation are as shown in Table 2.

**Table 5:** Transmural gradient in circumferential strain between endocardial and epicardial layers (G_endo-epi_), and longitudinal strain between the base and apex of the left ventricle (G_base-apex_).

	Total (n = 130)	Female (n = 70)	Male (n = 70)	P (M vs. F)
G_endo-ep_	6.9 ± 1.3	6.7 ± 1.4	7.1 ± 1.2	0.054
G_base-apex_	10.8 ± 5.6	11.2 ± 5.6	10.2 ± 5.6	0.313

All strain values are given in % and presented as the mean ± SD.

Gbase-apex: longitudinal gradient from the base to the apex of longitudinal strain.

Gendo-epi: transmural gradient from the endowall to the epiwall of circumferential strain.

**Table 6:** Intra- and inter-observer reproducibility for measurement of layer-specific strain (n = 26).

		Intra-observer			Inter-observer		
		Mean bias ± SD	ICC	CV (%)	Mean bias ± SD	ICC	CV (%)
Err	myo	0.01 ± 0.78	0.99	1.33	0.19 ± 1.93	0.96	3.32
	endo	0.14 ± 1.56	0.98	2.93	−0.38 ± 3.47	0.88	6.44
	mid	0.02 ± 0.91	0.99	1.44	0.29 ± 1.90	0.97	3.03
	epi	0.16 ± 1.16	0.99	1.86	0.12 ± 1.84	0.97	2.95
Ecc	myo	−0.19 ± 0.54	0.95	2.32	0.23 ± 0.81	0.88	3.38
	endo	−0.14 ± 0.78	0.95	2.57	0.07 ± 1.18	0.87	3.87
	mid	−0.15 ± 0.62	0.94	2.50	0.17 ± 0.91	0.86	3.63
	epi	−0.26 ± 0.67	0.87	3.55	0.45 ± 0.74	0.83	4.27
Ell	myo	0.24 ± 0.82	0.93	4.04	0.06 ± 1.02	0.89	4.86
	endo	0.22 ± 0.71	0.95	3.35	−0.22 ± 1.18	0.87	5.44
	mid	0.28 ± 0.79	0.93	3.91	−0.10 ± 1.04	0.89	4.90
	epi	0.17 ± 0.77	0.93	3.91	0.04 ± 0.98	0.89	4.91

Err = radial strain; Ecc = circumferential strain; Ell = longitudinal strain; ICC = intraclass correlation coefficient; CV = coefficient of variation; myo = myocardium; endo = endowall; mid = midwall; epi = epiwall.
