# Author Correction: **Distribution pattern of leftventricular myocardial strain analyzed by a cine MRI based deformation registration algorithm in healthy Chinese volunteers**

**DOI:** 10.1038/s41598-020-77822-4

**Published:** 2020-11-23

**Authors:** Hong Liu, Dan Yang, Ke Wan, Yong Luo, Jia-Yu Sun, Tian-Jing Zhang, Wei-Hao Li, Andreas Greiser, Marie-Pierre Jolly, Qing Zhang, Yu-Cheng Chen

**Affiliations:** 1 Cardiology Division, West China Hospital, Sichuan University, Chengdu, Sichuan Province China; 2 Radiology Department, West China Hospital, Sichuan University, Chengdu, Sichuan Province China; 3 Siemens Healthineers Northeast Asia Collaboration, Beijing, China; 4 Siemens Healthineers, Erlangen, Germany; 5 Siemens Healthineers, Medical Imaging Technologies, Princeton, NJ USA

Correction to: *Scientific Reports* 10.1038/srep45314, published online 28 March 2017

This Article contains an error in Table 5.

In the first row heading, the number of male patients,

“Male (n = 70)”.

should read:

“Male (n = 60)”.

